# Editorial for Future Trends in Ultra-Precision Machining

**DOI:** 10.3390/mi17040422

**Published:** 2026-03-30

**Authors:** Changlin Liu, Yanbin Zhang, Xiaoliang Liang

**Affiliations:** 1 School of Mechanical Science and Engineering, Huazhong University of Science and Technology, Wuhan 430074, China; 2 School of Mechanical and Automotive Engineering, Qingdao University of Technology, Qingdao 266520, China; 3 School of Mechanical Engineering, Shandong University, Jinan 250061, China

## 1. Introduction for This Special Issue of Future Trends in Ultra-Precision Machining

Ultra-precision machining (UPM) is an advanced manufacturing technology used to produce components with exceptionally high dimensional accuracy, surface integrity, and form precision [1,2]. In general, it refers to machining processes capable of achieving tolerances in the sub-micrometer range and surface roughness at the nanometer level, which forms the backbone and support of today’s innovative technology industries from optoelectronics, aerospace, optics, and biomedical engineering [3,4].

With the rising demand for precision devices in harsh environments, achieving UPM on brittle materials has become of great interest in research of advanced manufacturing. During machining, surface defects and subsurface damage such as cracks, fractures, and tool wear caused by their brittle nature is the main reason behind the deterioration of the machined surface quality of brittle materials [5,6,7]. Scholars have revealed that defect-free surfaces on brittle materials can be fabricated via ductile machining by decreasing the material removal thickness to a nanoscale level [8], while the machining mechanism is not fully revealed since microstructures such as crystal orientation and grain boundaries have significant influence on deformation behavior.

To overcome the undesirable machining defects and improve the machineability of brittle materials, various field-assisted machining technologies have been proposed, which introduce controllable external energy fields (such as laser heating [9], ultrasonic tool vibration [10], magnetic fields [11], etc.) to regulate the local mechanical response of the material or the removal mode of the machining process, ultimately improving the machined surface quality and machining efficiency. Furthermore, to amplify the effects of applied assistive fields, researchers have coupled the energy fields and explored the mechanisms in multi-field-assisted cutting [12]. In field-assisted cutting, the introduced energy field alters both the intrinsic properties of materials and the tool–workpiece interaction. Consequently, the underlying mechanisms differ fundamentally from those of conventional processes and single-field-assisted machining, making this a prominent research hotspot in the current field of UPM.

In recent years, Artificial Intelligence (AI) has become a pivotal core technology to promote the intelligent transformation of ultra-precision machining systems, aiming to address challenges such as highly coupled process interactions, nanometer-level precision requirements, limited process observability, and the high cost caused by trial-and-error experiments [13,14,15]. It is worth noting that digital twin technology enables a closed-loop integration including state perception, process prediction, and decision optimization, thereby providing system-level understanding and control for complex machining processes. At the same time, the rapid advancement of foundation models, such as Large Language Models [16], Vision Foundation Models [17] and Industrial Time-series Foundation Models [18], has provided new technical approaches for manufacturing knowledge modeling, cross-process knowledge transfer, and human–machine collaborative decision-making. Compared to traditional methods that often rely on empirical parameter tuning, simplified analytical models, or process-specific modeling, these developments are expected to fundamentally enhance process predictability, controllability, and system-level intelligence in next-generation ultra-precision manufacturing systems.

**Figure 1:**
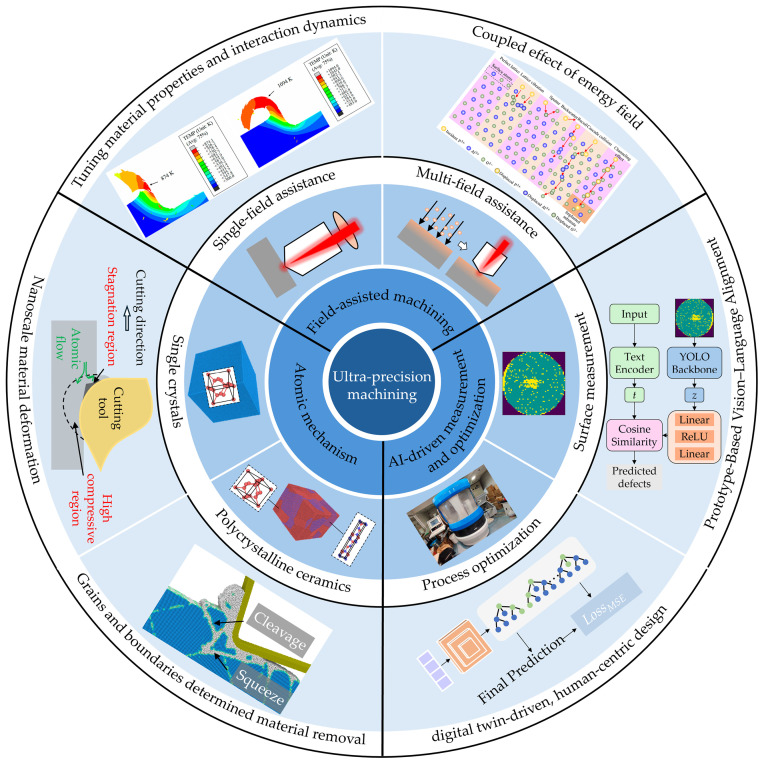
Topics covered in the Special Issue titled “Future trend in Ultra-precision machining”.

Building on sincere cooperation with the Guest Editors, this Special Issue concentrates on research in the field of ultra-precision machining, including removal mechanisms, field-assisted machining, and AI-driven process optimization and measurement. As depicted in Figure 1, this Special Issue featured a diverse array of topics, publishing a total of 8 contributions, comprising 7 original research articles and 1 review paper, which is briefly introduced below according to three aspects (i.e., atomic mechanism, field-assisted machining, AI-driven measurement and process optimization) of UPM in this Special Issue.

## 2. Material Removal Mechanism of UPM in This Special Issue

The deformation mechanism of material in UPM differs from that in macroscale machining since the material removal thickness decreases into nanoscale, which is comparable to the cutting tool edge radius. Furthermore, microstructures such as grain boundaries and crystal orientation have significant influence on the material removal behavior. Due to the difficulty in direct observation of material deformation at the nanoscale level, molecular dynamics (MD) simulation [19,20], which is based on first-principles calculations or empirical interatomic potentials, has been widely used to describe the material deformation behavior in UPM owing to its advantages in simulating plastic deformation behavior such as shear flow [21], dislocation propagation [22], and phase transition [23]. The material removal mechanism of UPM covered in this Special Issue includes investigation and review of the deformation mechanisms of different brittle materials including single crystals, polycrystal ceramics, and amorphous glass.

For typical brittle materials, Sheng et al. [24] summarized previous research on the machining mechanism of brittle materials during single-point diamond turning and synthesized numerical and experimental findings across material classes, including single crystals, polycrystalline ceramics, and amorphous glass. They discussed the material removal mechanisms represented by ductile, transition, and brittle removal.

Differing from single crystals, the material removal mechanism in polycrystalline materials (such as reaction-bonded silicon carbide) is determined by grain distribution and boundaries. Mo et al. [25] conducted MD to investigate the effect of abrasive size on surface morphology and subsurface deformation mechanism of reaction-bonded silicon carbide during nano-grinding. They analyzed the surface swelling, material removal rate, and high-pressure phase transition under different grain sizes. These results provided the theoretical basis for an in-depth understanding in mechanisms during UPM of materials with random distribution of grains.

## 3. Field-Assisted Machining in This Special Issue

This Special Issue covers a wide range of investigations into single-field-assisted machining including laser-assisted cutting, and multi-field-assisted machining (e.g., laser-vibration assisted cutting, implantation-laser assisted cutting).

For brittle materials such as glass-ceramic optical components, the high-hardness and low-fracture toughness make it prone to cracks and subsurface damage during conventional cutting. Li et al. [26] investigated the dynamic removal mechanisms of glass–ceramics under laser-assisted nanoscale cutting conditions through numerical simulations and systematic experiments. They quantified the enhancement effect of laser power on the critical depth of no observed surface cracks (NOSC) and revealed the mechanisms through which laser assistance inhibits crack propagation. The findings provide theoretical support for optimizing laser-assisted cutting parameters and achieving high-quality machining of glass–ceramics.

For sapphire crystals, which have extraordinary hardness and are widely used in advanced optics, microelectronic devices, and medical instruments. The improvement in machineability by a single assistive field is limited. Ke et al. [27] used ion implantation and laser-assisted cutting to improve the machining performance of C-plane sapphire. Their groove cutting experiments verified the enhancement in ductile machinability of the modified sapphire under LADM. At a laser power of 16 W, the ductile–brittle transition depth of the modified sapphire increased to 450.67 nm, representing a 51.57% improvement over conventional cutting. The findings of this study provide valuable insights for improving the ductile machining performance of hard and brittle materials.

During multi-field-assisted machining, the machining mechanism is complex since multiple interactions are involved in the machining process. Chu et al. [28] investigated the machining mechanism of single-crystal silicon under the combination of laser heating and tool vibration using MD simulations. They discussed the effect of tool vibration trajectory determined by different tool edge radii is discussed under the condition of raising temperature. Their results help to improve the understanding of machining mechanics in multi-field-assisted machining.

## 4. AI-Driven Measurement and Process Optimization in Special Issue

This Special Issue focuses on Artificial Intelligence (AI)-based surface defect measurement [16] and process optimization of the ultra-precision machining, aiming to effectively control the production process, reduce variation in the manufacturing process, and improve the yield rate represent important competitive factors for wafer factories.

With the rapid development of semiconductor manufacturing technology, methods to wafer bin maps provide valuable information for engineers to quickly identify potential root causes through accurate pattern recognition. Wang et al. [16] proposed YOLO-LA, a lightweight prototype-based vision–language alignment framework that integrates a pretrained frozen YOLO backbone with a frozen text encoder to enhance wafer defect recognition. They introduced a learnable projection head to map visual features into a shared embedding space, enabling classification through cosine similarity. The proposed framework is lightweight and suitable for real-time industrial wafer inspection systems.

During UPM, maintaining the machining quality presents a greater challenge when working conditions change due to the nanoscale material removal and surface roughness requirement. Mo et al. [29] conceptually proposed a digital twin (DT)-driven, human-centric design framework that integrates key factors of multi-jet polishing process. They introduced a feature-encoded transfer learning-based model to enhance surface roughness prediction accuracy and robustness under varying working conditions. Their model offers a practical and extensible perspective for optimizing complex ultra-precision manufacturing processes under data-scarce and uncertainty-dominated conditions.

With the continuous development of semiconductor manufacturing technology and information technology, the sizes of wafer chips are becoming smaller and the variety is increasing, which has put forward high requirements for wafer chip precision manufacturing and packaging workshops. Wang et al. [30] researched the wafer chip precision packaging workshop rescheduling problem under events of machine breakdown, emergency order inserting, and original order modification. They proposed a mathematical model for the addressed problem and a hybrid algorithm combining an improved firefly optimization framework. Their model is effective and stable and is superior to the current advanced algorithms.

To conclude, we would like to acknowledge all the authors for their contributions to the success of this Special Issue in *Micromachines*, as well as the reviewers whose feedback helped to improve the quality of the published papers.
